# Gibberellin-mediated RGA-LIKE1 degradation regulates embryo sac development in Arabidopsis

**DOI:** 10.1093/jxb/eraa395

**Published:** 2020-08-26

**Authors:** Maria Dolores Gomez, Daniela Barro-Trastoy, Clara Fuster-Almunia, Pablo Tornero, Jose M Alonso, Miguel A Perez-Amador

**Affiliations:** 1 Instituto de Biología Molecular y Celular de Plantas (IBMCP), Universidad Politécnica de Valencia (UPV)–Consejo Superior de Investigaciones Científicas (CSIC), Ciudad Politécnica de la Innovación, Ed. 8E, Ingeniero Fausto Elio s/n, Valencia, Spain; 2 Department of Plant and Microbial Biology, Program in Genetics, North Carolina State University, Raleigh, NC, USA; 3 Trinity College Dublin, Ireland

**Keywords:** Arabidopsis, DELLA, development, embryo sac, gibberellin, megagametogenesis, ovule, RGL1

## Abstract

Ovule development is essential for plant survival, as it allows correct embryo and seed development upon fertilization. The female gametophyte is formed in the central area of the nucellus during ovule development, in a complex developmental programme that involves key regulatory genes and the plant hormones auxins and brassinosteroids. Here we provide novel evidence of the role of gibberellins (GAs) in the control of megagametogenesis and embryo sac development, via the GA-dependent degradation of RGA-LIKE1 (RGL1) in the ovule primordia. *YPet-rgl1Δ17* plants, which express a dominant version of RGL1, showed reduced fertility, mainly due to altered embryo sac formation that varied from partial to total ablation. *YPet-rgl1Δ17* ovules followed normal development of the megaspore mother cell, meiosis, and formation of the functional megaspore, but *YPet-rgl1Δ17* plants had impaired mitotic divisions of the functional megaspore. This phenotype is RGL1-specific, as it is not observed in any other dominant mutants of the DELLA proteins. Expression analysis of *YPet-rgl1Δ17* coupled to *in situ* localization of bioactive GAs in ovule primordia led us to propose a mechanism of GA-mediated RGL1 degradation that allows proper embryo sac development. Taken together, our data unravel a novel specific role of GAs in the control of female gametophyte development.

## Teaser

New genetic tools to study RGL1 expression and function through plant development allowed the uncovering of an RGL1-specific role in the control of female gametophyte development.

## Introduction

Ovule development is a key process in the perpetuation of plant species, as it ensures the correct formation of the female gametophyte and the subsequent embryo and seed development upon fertilization. Ovule primordia initiation and growth have been studied mainly in the model species Arabidopsis (Schneitz *et al.*, 1995, 1997; Lora *et al.*, 2016; Pinto *et al.*, 2019; Cucinotta *et al.*, 2020), for which detailed developmental stages have been defined (Schneitz *et al.*, 1995).

Ovule primordia, composed solely of diploid cells, emerge from the placental tissue as finger-like protrusions from the placenta in the medial domain of the developing ovary. Successive cell divisions give rise to three prominent domains along a proximal–distal axis: the funiculus, which connects the ovule to the placenta; the chalaza in the central domain, which gives rise to the inner and outer integuments; and the nucellus in the distal region, which produces a single germline cell, the megaspore mother cell (MMC), the progenitor of a single haploid functional megaspore (FM) (Pinto *et al.*, 2019). At early phases of ovule development, two cell layers, epidermal and subepidermal, can be distinguished in the nucellus. The most distal cell in the subepidermal layer will become the germline upon differentiation into an archesporial cell that later expands to form the MMC (stage 2-I). Meiosis of the MMC produces four haploid megaspores of which only one remains as the FM (stage 3-I). Once established, the FM undergoes megagametogenesis, a series of transformation processes to generate the mature female gametophyte or embryo sac. This developmental process includes three rounds of mitotic divisions, reorganization of nuclei along the embryo sac, vacuole biogenesis, as well as cellular differentiation to ensure female gametophyte fertilization and, therefore, plant reproduction.

Several plant hormones have been shown to be essential for mitosis progression and vacuole formation during the formation of the female gametophyte. In Arabidopsis, mutations in several genes cause mitotic arrest at different embryo sac developmental stages (Serbes *et al.* 2019). These include (i) *PIN-FORMED1* (*PIN1*), *AUX1*, and *LIKE AUX1* (*LAX1*) genes, which mediate transport of auxin from the sporophytic tissue into the embryo sac; (ii) *YUCCA8* (*YUC8*) and *TRYPTOPHAN AMINOTRANSFERASE OF ARABIDOPSIS1* (*TAA1*) genes, necessary for auxin synthesis; and (iii) *CYP851*, which encodes a brassinosteroid synthesis enzyme. Therefore, auxin and brassinosteroid phytohormones are necessary for proper female gametophyte development.

We have reported that gibberellins (GAs) play a major role in both ovule primordia initiation (Gomez *et al.*, 2018, 2019) and ovule development (Gomez *et al.*, 2016). In both cases, constitutive GA signalling impairs these processes. DELLA proteins, a family of plant-specific GRAS transcriptional regulators, are central components of the GA signalling pathway, acting as negative regulators that block a large array of GA-mediated developmental processes essential for the plant life cycle (Sun, 2011; Davière and Achard, 2013, 2016; Hedden and Sponsel 2015; Vera-Sirera *et al.*, 2015). Upon binding to the GA receptor GID1, bioactive GAs mediate the polyubiquitination and the 26S proteasome-dependent degradation of DELLA proteins. Therefore, GAs act by modulating the degradation of DELLA proteins. At low levels of GA, DELLA proteins are stable, allowing the GA response to be blocked, whereas GA synthesis mediates DELLA removal and allows GA responses to take place.

The so-called DELLA domain lies in the N-terminal part of the protein (Dill *et al.*, 2001; Vera-Sirera *et al.*, 2015), and removal of this domain results in a stable GA-resistant protein that constitutively blocks the GA response. Whereas most plant species encode only one or two DELLA proteins, the Arabidopsis genome encodes up to five *DELLA* genes: *GAI* (*GA-INSENSITIVE*, *At1g14920*), *RGA* (*REPRESSOR OF GA1-3*, *At2g01570*), *RGL1* (*RGA-LIKE1*, *At1g66350*), *RGL2* (*At3g03450*), and *RGL3* (*At5g17490*). The presence of multiple DELLA proteins raises an important question regarding the degree of functional redundancy versus specificity of each DELLA in Arabidopsis (Gallego-Bartolomé *et al.*, 2010; Sun, 2011; Vera-Sirera *et al.*, 2015). During ovule development, several DELLA proteins have been shown to act redundantly as positive factors. GAI, RGA, and RGL2 participate in ovule primordia initiation, and GAI, RGA, RGL1, and RGL2 co-ordinately regulate integument development (Gomez *et al.*, 2016, 2018). On the other hand, the GA receptor GID1 has been implicated in the regulation of the fusion of central cell nuclei in the female gametophyte just before fertilization (Gomez *et al.*, 2018) and in the correct differentiation of a single MMC (Ferreira *et al.*, 2018).

Genetic and molecular tools are key for correctly assigning function to a particular gene. In the case of the *DELLA* genes, gain-of-function mutant alleles have been fundamental to uncovering their molecular and physiological function. These mutants were generated by removing the conserved DELLA domain to prevent GA-dependent protein degradation, and these truncated genes were then expressed under the control of the corresponding endogenous promoter, as is the case of *gai-1*, *GFP-rgaΔ17*, or *YPet-rgl2Δ17* (Koorneef *et al.*, 1985; Peng *et al.*, 1997; Dill *et al.*, 2001; Gomez *et al*., 2019). No similar line has been available for RGL1, however. Wen and Chang (2002) reported a dominant RGL1 line carrying a deletion of the DELLA domain, similar to that of *gai-1*, whose expression was controlled by the cauliflower mosaic virus (CaMV) 35S promoter. Plants expressing the *35S:rgl1Δ17* construct were dark green, dwarf, with underdeveloped and stunted flowers. The use of CaMV rather than an endogenous promoter impedes conclusion on whether the phenotypes observed are truly related to the activity of the native RGL1 protein.

To get a deeper insight in the role of RGL1 in ovule development, we generated translational fusion lines that express YPet-tagged versions of either the native RGL1 (*pRGL1:RGL1-YPet*) or a dominant version with a 17-aa DELLA domain deletion (*pRGL1:YPet-rgl1Δ17*), both controlled by endogenous *RGL1* regulatory sequences. These lines provide bona fide tools to study the participation of RGL1 in a wide variety of plant developmental processes regulated by GAs, and to uncover new unknown functions. Here we confirm that RGL1 controls organ elongation, as in the inflorescence stems, flower whorls, and siliques. Moreover, RGL1 participated in the control of ovule development, by impairing the formation of the embryo sac. Interestingly, dominant versions of GAI, RGA, or RGL2 did not show embryo sac defects, pointing to RGL1 as the only DELLA protein that acts as a specific negative regulator of embryo sac development. Finally, *in situ* accumulation of bioactive GAs in ovule primordia correlated with *YPet-rgl1Δ17* expression. In summary, GAs participate in the control of female gametophyte development via the GA-mediated degradation of RGL1 in the ovule.

## Materials and methods

### Plant material

Arabidopsis plants from the Landsberg *erecta* (L*er*) genetic background were used. Dominant mutants g*ai-1*, *GFP-rgaΔ17*, and *YPet-rgl2Δ17* were described previously (Peng *et al.*, 1997; Dill *et al.*, 2001; Gomez *et al*., 2019). The *rgl1-1* null mutant was obtained from the Nottingham Arabidopsis Stock Center (www.arabidopsis.info). GA hormone-activated Cas9-based repressor (HACR) plants (Khakhar *et al.*, 2018) were provided by Dr J. L. Nemhauser (University of Washington, USA). Seeds were surface sterilized in ethanol and plated onto ½MS medium plates (Murashige and Skoog, 1962). Plates were kept at 4 °C in darkness for 4 d and were moved to a growth chamber at 22 °C under a long-day (LD) photoperiod (16 h–8 h) for 10 d. Seedlings were then transferred to soil (a mixture of peat moss, vermiculite, and perlite, in a ratio of 2:1:1) and grown to maturity in a growth chamber at 22 °C under the LD photoperiod. MS media were supplemented with 5 µM ammonium glufosinate to select transgenic plants. To induce DELLA degradation, seedlings were placed on top of sterile filter paper and transferred to plates supplemented with 1 µM GA_4 + 7_ for 24 h before confocal microscopy analysis.

### Flowering time, determination of ovule number, and fertility assays

For the flowering time assay, seeds were directly sown on pots and maintained under either LD or short day (SD; 8 h–16 h) photoperiods in controlled growth chambers at 22 °C. Flowering time was determined as the number of total leaves formed at the time of bolting. The number of days to bolting was also scored. Three biological replicates were used, for a total of ~120 plants. Plant height was scored by measuring the length of the main inflorescence stem in mature pants (*n*>30). Ovule number was determined as described in Gomez *et al.* (2018), and ovary size was determined in the same pistils used for ovule number determination, from images taken under a stereomicroscope (*n*=10–12). For the fertility analysis, flower buds of L*er* or *YPet-rgl1Δ17* were hand-emasculated 1 d before anthesis, and pistils were hand-pollinated the next day with mature pollen from either L*er* or *YPet-rgl1Δ17* plants. In each plant only one flower, number 10–15 in the inflorescence, was used. Fruits were collected at maturity and seed number and silique length were measured (*n*≥30 per pollination). All experiments were repeated three times, with similar results.

### Construction of *pRGL1:RGL1-YPet* and *pRGL1:YPet-rgl1Δ17*

Translational fusions of YPet with RGL1 and a dominant version rgl1Δ17 were generated from a genomic clone by bacterial homologous recombination technology (recombineering), basically as described in Brumos *et al.* (2020). Briefly, both constructs were generated using the JAtY clone JAtY50E24 from the JIC (JAtY library, https://abrc.osu.edu/stocks/number/CD4-96) in the pYLTAC17 vector, which contains the *RGL1* locus (*At1g66350*) located at 66.8 kb of the 80.3 kb genomic fragment. Bacterial media were supplemented with 25 µg ml^−1^ kanamycin plus the corresponding antibiotic, as indicated. All oligonucleotides used are listed in the Supplementary Table S1 at *JXB* online, and the general procedure is described in Supplementary Figs S1–S3. First, the JAtY clone was moved from DH10B to the SW105 *Escherichia coli* strain to carry out the recombineering steps. The YPet tag protein (Zhou *et al.*, 2011) was introduced in-frame at the Nt or Ct of *RGL1* to generate *pRGL1:YPet-RGL1* and *pRGL1:RGL1-YPet*, using a YPet-FRT-Amp cassette (Brumos *et al.*, 2020). The ampicillin resistance gene was then removed by FRT-mediated recombination, and constructs were confirmed by sequencing.

For the elimination of the DELLA domain in *pRGL1:YPet-RGL1*, first an RPSL-Amp cassette was introduced to replace the 51 bp of *RGL1* that encode the 17-aa DELLA domain DELLVVLGYKVRSSDMA, equivalent to that of *gai-1* (Peng *et al.*, 1997), *GFP-rgaΔ17* (Dill *et al.*, 2001) or *pRGL2:YPet-rgl2Δ17* (Gomez *et al*., 2019), and positive recombinants were selected via ampicillin. The RPSL-Amp cassette was removed by recombination with a PCR product generated with oligos delF and delR (see Supplementary Fig. 2C) using the original TAC clone as a template. Oligo delF corresponds to 38 nt upstream and 22 nt downstream of the DELLA region, while the delR oligo is located between 179 and 202 nt downstream of the DELLA region. Therefore, the 240 nt PCR product does not contain the DELLA region but extends from −38 to + 202 of this region. Positive colonies were selected in streptomycin, as the presence of RPSL confers sensitivity to the antibiotic, and the construct was confirmed by sequencing.

The constructs were transferred to a recA-deficient *Agrobacterium tumefaciens* GV3101 (pMP90) strain (Zhou *et al.*, 2011) and L*er* Arabidopsis plants were transformed by the floral dip method (Clough and Bent, 1998). Transgenic plants were selected in ammonium glufosinate, and T3 homozygous lines segregating as a single locus were selected.

### Histological procedures

Ovule morphology was studied using chloral hydrate clearing and differential interference contrast light microscopy according to Weigel and Glazebrook (2002). Images were recorded using a Nikon Eclipse E600 microscope equipped with a Nikon DS-Ri1 digital camera. The number of ovules with a wild-type (WT)-like shape or mild and severe defects in embryo sac development was determined from a sample of 875 mature ovules of emasculated flowers from 16 *YPet-rgl1Δ17* pistils, each from an individual plant.

For histological analysis of ovule development, L*er* and *YPet-rgl1Δ17* inflorescences were fixed overnight in FAE (5% (v/v) formaldehyde, 10% (v/v) acetic acid, 50% (v/v) ethanol), dehydrated in a 50, 70, 90, and 100% (v/v) ethanol series, embedded in Technovit 7100 resin, sectioned in a Reichert Jung Ultracut E microtome at 3 μm, and stained in 0.02% Toluidine blue as described in Gomez *et al*. (2004). Images were captured with a Leica DM5000 microscope.

### *In situ* RNA hybridization

Arabidopsis inflorescences were embedded in paraffin, sectioned, and hybridized as described by Gomez *et al.* (2018). The RGL1 template was amplified (forward primer: GAATCAAGCGATACTTGAGG; reverse primer: CATTTCATTGGCCTGACCCTG) and cDNA was cloned into the pGem-T Easy vector (Promega). Sense and antisense probe were synthesized using the corresponding SP6 and T7 RNA polymerases in the vector. Control experiments were performed with sense probes and no significant signal was detected. Images were recorded using a Nikon Eclipse E600 microscope equipped with a Nikon DS-Ri1 digital camera.

### Confocal laser scanning microscopy

Confocal laser scanning microscopy (CSLM) was used to analyse the development of the different cellular layers that make up the *YPet-rgl1Δ17* ovules. For this, inflorescences were fixed with 4% paraformaldehyde for 1 h with vacuum treatment. After fixation, the samples were washed twice for 1 min in 1× phosphate-buffered saline, moved to ClearSee solution (Kurihara *et al.*, 2015) and cleared for 1 week at room temperature. After clearing, the inflorescences were stained with Calcofluor White as described by Ursache *et al.* (2018). To detect and image bound Calcofluor White, we used a Zeiss LSM 780 confocal microscope with excitation at 405 nm and detection at 425–475 nm. The distribution of RGL1–YPet and YPet–rgl1Δ17 proteins during ovule development was studied with the same confocal microscope, with excitation at 514 nm and emission filters set to 520–540 nm. Finally, the *in situ* localization of bioactive GAs in the GA HACR plants were analysed by the detection of Venus fluorescent protein with excitation at 488 nm and detection at 510–530 nm. The identity of fluorescence signals was confirmed with a λ-scan.

## Results and Discussion

### Construction of *pRGL1:RGL1-YPet* and *pRGL1:YPet-rgl1Δ17* transgenic lines

The availability of bona fide reporter lines is crucial to assess the proper expression pattern of a gene of interest and to correlate it to the molecular function. We generated a translational fusion reporter line of RGL1 fused to the fluorescent protein YPet at the Ct and Nt, using a recombineering strategy (see Supplementary Figs S1–S3 and ‘Materials and methods’ section for details). In addition, a gain-of-function allele of RGL1 (*pRGL1:YPet-rgl1Δ17*) was generated by deleting the 17-aa DELLA domain (DELLVVLGYKVRSSDMA) located at position 32–48 of the YPet–RGL1 protein also by recombineering (Supplementary Fig. S4); elimination of this domain should prevent GA-mediated degradation of the YPet–rgl1Δ17 protein. After trimming both genomic clones to improve stability during plant transformation, the final constructs included genomic sequences 10 kb upstream and 5 kb downstream of the *RGL1* locus (Supplementary Fig. S5), which potentially contain all the regulatory regions, providing a reliable expression pattern likely to reflect that of the native gene. Transgenic plants were generated for both *pRGL1:RGL1-YPet* and *pRGL1:YPet-rgl1Δ17* constructs. Different lines for each construct showed similar phenotypes; therefore, single lines (thereafter *RGL1-YPet* and *YPet-rgl1Δ17*) were selected for further analysis.

### RGL1–YPet is degraded by GAs, but YPet–rgl1Δ17 is GA-resistant

**Fig. 1.**
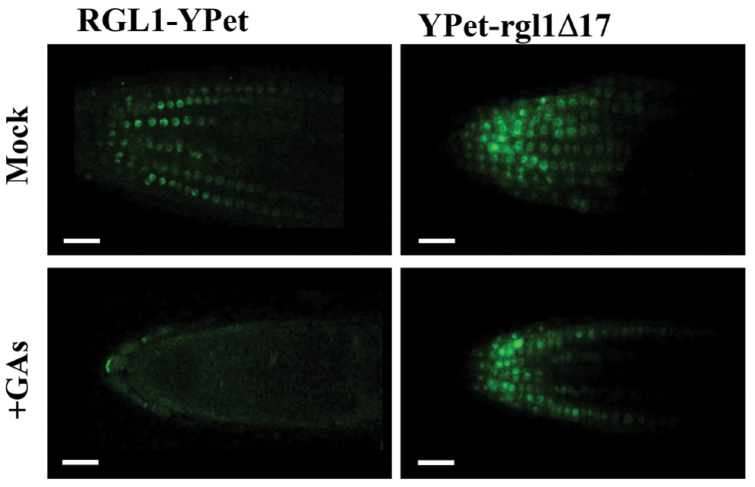
GA-mediated degradation of RGL1–YPet but not YPet–rgl1Δ17. RGL1–YPet and YPet–rgl1Δ17 proteins were visualized in the root tips of 4-day-old seedlings of Arabidopsis transgenic lines *pRGL1:RGL1-YPet* and *pRGL1:YPet-rgl1Δ17*, respectively (Mock, upper panels). RGL1–YPet but not YPet–rgl1*Δ*17 was degraded in the presence of 1 µM GA_4 + 7_ (+GA, lower panels). Scale bars represent 20 µm. (This figure is available in colour at *JXB* online.)

The stability of the RGL1–YPet and YPet–rgl1Δ17 fusion proteins was analysed in primary roots of 4-day-old seedlings upon GA treatment (Fig. 1). Both RGL1–YPet and YPet–rgl1Δ17 were located at the cell division zone of the primary root, the levels of the dominant YPet–rgl1Δ17 being much higher than those of the protein containing the DELLA domain. In addition, tagged proteins were located in the nucleus of the root cells as was previously reported for RGL1 and other DELLA proteins (Silverstone *et al.*, 2001; Fleck and Harberd, 2002; Wen and Chang, 2002; Gomez *et al*., 2019). Moreover, treatment with GAs promoted a strong degradation of RGL1–YPet, whereas levels of the dominant version YPet–rgl1Δ17 remained nearly identical to those of the untreated plants. Therefore, the dominant GA-resistant version, YPet–rgl1Δ17, blocked RGL1-dependent GA signalling.

Strikingly, whereas nuclear-localized RGL1–YPet protein can be degraded by GAs, no RGL1 protein degradation was observed using a green fluorescent protein (GFP)-fused RGL1 protein under the control of the strong CaMV 35S promoter (Wen and Chang, 2002). This discrepancy may reflect the differences in promoter activities. Similar to the *35:GFP-RGL1* line, degradation of the 35S-driven GAI–GFP fusion protein by GAs was also not detectable (Fleck and Harberd, 2002).

### *YPet-rgl1* *Δ* *17* plants uncover RGL1-dependent growth functions

We generated YPet-tagged versions of RGL1 that include the 16.5 kb genomic region around the *RGL1* locus, including 10 kb of the promoter and a 5-kb downstream region that most probably directs the expression of the fusion proteins in a similar manner to the native RGL1. In addition, the dominant YPet–rgl1Δ17 protein was GA-resistant, blocking the RGL1-dependent GA-mediated development. Therefore, the phenotypes of the dominant line are most probably the consequence of specifically blocking RGL1-dependent GA responses, uncovering the functions of RGL1 in plant development.

**Fig. 2.**
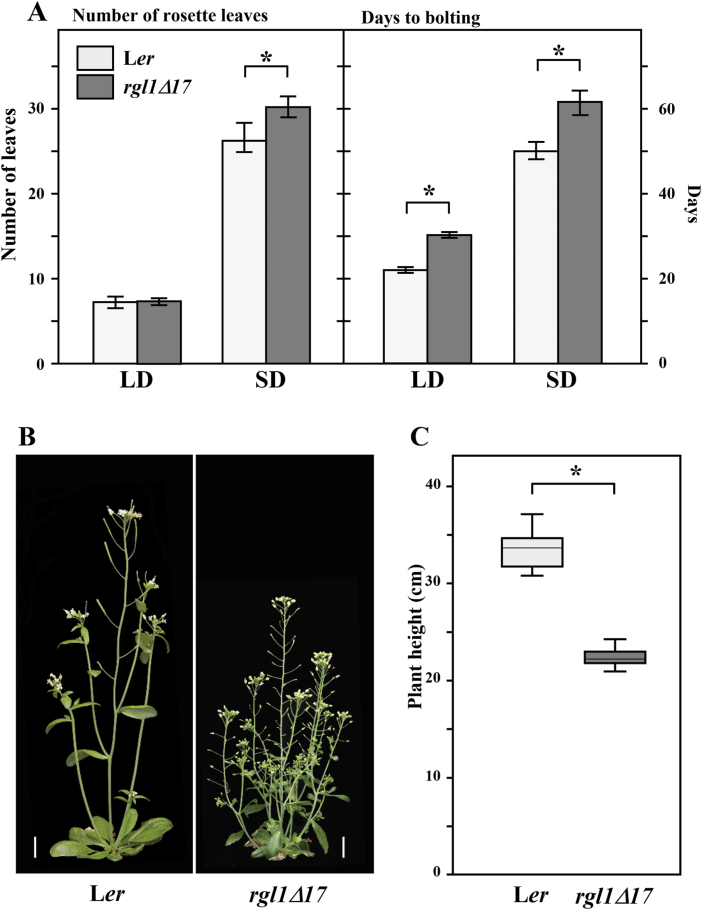
Expression of YPet–rgl1Δ17 delayed flowering and reduced plant height. (A) Number of rosette leaves per plant produced (left) or days (right) from seed germination to bolting in L*er* and *YPet-rgl1Δ17* (*rgl1Δ17* thereafter in the figures) in Arabidopsis plants grown under long (LD, 16h light–8h dark) or short (SD, 8h light–16h dark) day. (B) Image of mature L*er* and *YPet-rgl1Δ17* plants. (C) Quantification of plant height of mature L*er* and *YPet-rgl1Δ17* plants. Significant differences in (A, C) (Student’s *t*-test analysis) between L*er* and *YPet-rgl1Δ17* are marked (*P-value<0.01). In (A), data shown are the mean and SE from three biological replicas (*n*=37–42, per replica), and in (C) data are the mean and SD (*n*>30). Scale bars in (B) represent 1 cm. (This figure is available in colour at *JXB* online.)

**Fig. 3.**
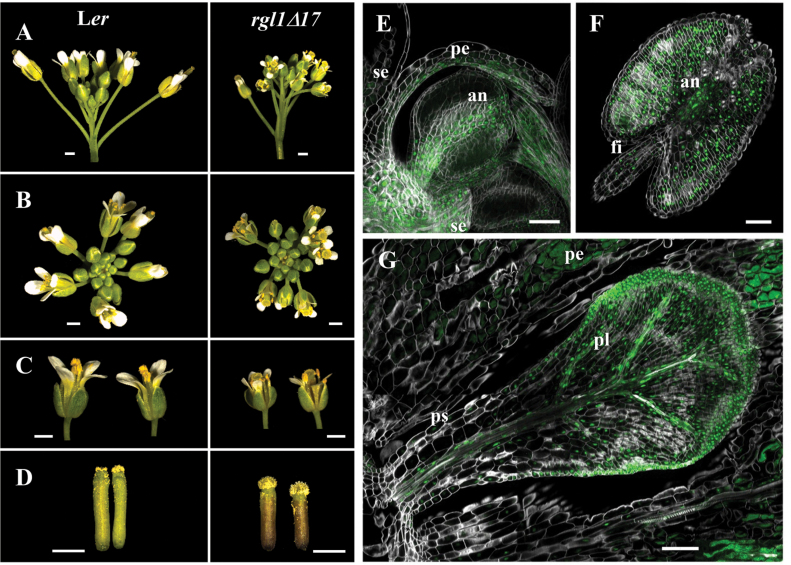
Expression of YPet–rgl1Δ17 promoted alterations in inflorescences, flowers, and pistils. (A–D) Images of L*er* and *YPet-rgl1Δ17* inflorescences in lateral (A) or zenithal view (B), flowers at anthesis (C), and pistils at anthesis (D). (E–G) CLSM images of expression of YPet–rgl1*Δ*17 in the sepal, petal, and a young anther (E), mature anther (F), and developing petal (G) of *YPet-rgl1Δ17* plants. Scale bars represent 1 mm in (A–D), 40 µm in (E), and 50 µm in (F–G). an, anther; fi, stamen filament; pe, petal; pl, petal lamina; ps, petal stalk; se, sepal. (This figure is available in colour at *JXB* online.)

At the vegetative level, *YPet-rgl1Δ17* plants showed delayed flowering and reduced plant height with shorter floral stems (Fig. 2A–C). Delayed flowering was most evident under SD conditions (i.e. 8 h–16 h regimen) when plants flowered after more rosette leaves were produced (Fig. 2A). Under LD conditions (16 h–8 h regimen), *YPet-rgl1Δ17* plants flowered 4 d later than the WT, with the same number of rosette leaves. Adult plant architecture was also modified by *YPet-rgl1Δ17*. These plants showed dwarfism, partial loss of apical dominance, and increased shoot branching (Fig. 2B, C). In addition, *YPet-rgl1Δ17* plants evidenced a darker green colour compared with L*er*. In terms of reproductive development, *YPet-rgl1Δ17* plants also showed morphological alterations, including compact inflorescences due to shorter flower petioles, and reduced floral size by the shortening of all four floral organs (Fig. 3A–D).

We next studied the expression of RGL1 using the YPet-tagged lines. RGL1–YPet protein was not detected in the different tissues analysed by CSLM, with the exception of the root tip, possibly due to its low abundance, as endogenous bioactive GAs would trigger its degradation to enable organ growth and development. Stable YPet–rgl1Δ17 protein was clearly visualized in a large variety of tissues, however. Therefore, localization of YPet–rgl1Δ17 protein was used to infer the expression pattern of RGL1 during floral organ development. Overall, reduction of floral organs was correlated with expression of *YPet-rgl1Δ17* (Fig. 3E–G). The chimeric protein was detected in sepals and petals, especially in the lamina. Expression was also apparent in the stamens, both in filaments at early stages and in anthers throughout development. Therefore, the limited size of floral organs is most probably due to blockage of growth imposed by the dominant YPet–rgl1Δ17 protein. These flower phenotypes were stable throughout plant development.

The data reported here support the participation of RGL1 in flowering, stem elongation, and floral organ development. Wen and Chang (2002) reported similar but enhanced phenotypes in a *35S:rgl1Δ17* line, which overexpresses a dominant version of RGL1 driven by the strong constitutive CaMV 35S promoter. These included severe dwarfism, dark pigmentation, and delayed flowering. But there were also remarkable differences between the *35S:rgl1Δ17* (Wen and Chang, 2002) and *YPet-rgl1Δ17* phenotypes. For example, in the 35S line, expression of *rgl1Δ17* in rosette leaves led to a strong reduction in rosette size similar to the GA-deficient *ga1*-*3* mutant. In contrast, no major defects in rosette leaves were observed in *YPet-rgl1Δ17* plants, which suggests that native RGL1 expression in the rosette is very low. The differences in the phenotype penetrance between *35S:rgl1Δ17* and *pRGL1:YPet-rgl1Δ17* lines are most probably caused by the different promoter used: the strong ectopic expression driven by the constitutive 35S promoter, compared with the *RGL1* endogenous regulatory sequences in the *pRGL1:YPet-rgl1Δ17* line.

An important issue regarding the role of the *DELLA* family in Arabidopsis is the degree of overlapping versus specific roles of each particular gene in the control of GA-mediated developmental processes (Sun, 2011). The participation of the different DELLA proteins in several developmental processes has been uncovered by using single and multiple loss-of-function mutants in different combinations (reviewed in Vera-Sirera *et al.*, 2015). An analysis of the phenotypes of plants upon RGA–RGL2 promoter switching suggested that functional diversification of DELLA proteins relies mainly on changes in their gene expression patterns rather than on their molecular function (Gallego-Bartolomé *et al.*, 2010). Therefore, temporal and spatial expression patterns of the different DELLA proteins may be the major contributor to their functions in development. In view of this, it is critical to use their endogenous regulatory sequence to get bona fide information regarding the role of *RGL1*, as is used in the case of the *pRGL1:YPet-rgl1Δ17* line.

### Seed number is reduced in *YPet-rgl1Δ17* plants

GAs participate in the regulation of ovule primordial formation (Gomez *et al.*, 2018) and in ovule integument development (Gomez *et al.*, 2016). We used *YPet-rgl1Δ17* plants to study the contribution of RGL1 to the regulation of ovule initiation and integument development but also to uncover new roles of this protein in ovule and seed development.

**Fig. 4.**
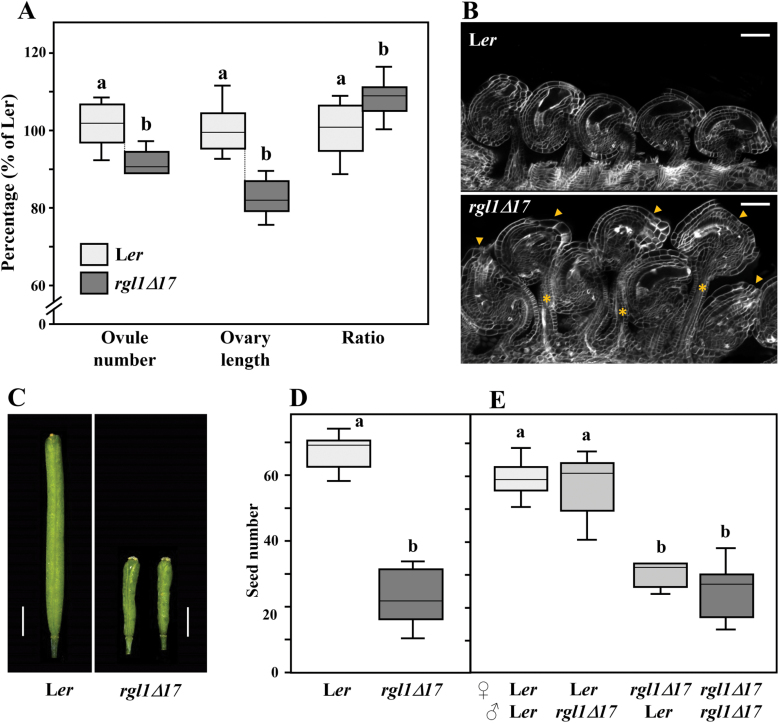
Ovule and seed number was altered in *YPet-rgl1Δ17* plants. (A) Ovule number per pistil, ovary length, and the ratio of ovule number to ovary length in flowers at anthesis of L*er* (light grey) and *YPet-rgl1Δ17* (dark grey) plants. (B) CLSM images of representative mature ovules of L*er* (upper panel) and *YPet-rgl1Δ17* (lower panel) plants. Asterisks mark long funiculi; arrowheads mark altered ovules in *YPet-rgl1Δ17*. (C) Images of mature self-pollinated fruits of L*er* and *YPet-rgl1Δ17* plants. (D) Number of seeds from self-pollinated fruits of L*er* and *YPet-rgl1Δ17* plants. (E) Number of seeds from cross-pollinated fruits of L*er* and *YPet-rgl1Δ17* plants. Data are represented as boxplots; *n*=10–12 in (A) and *n*≥30 in (D, E). Letters above each box indicate statistical significance as determined by an ANOVA and a Bonferroni *post hoc* test for multiple comparisons (*P*-value<0.01). Data that are not significantly different are marked with the same letter. Scale bars represent 50 µm in (B) and 2 mm in (C). (This figure is available in colour at *JXB* online.)

First, we scored ovule number, ovary length, and the ratio of ovule number to ovary length in *YPet-rgl1Δ17* plants and compared these with the L*er* WT (Fig. 4A). Expression of *YPet-rgl1Δ17* caused a small reduction in the number of ovules per pistil, but had a stronger effect in reducing ovary length, leading to an increase in ovule density within the ovary. As ovule initiation and pistil development take place at the same time, the ovule number alterations observed suggests that YPet–rgl1Δ17 mainly blocks ovary valve elongation, resulting in smaller pistils, similar to the shortening of other floral organs. The increased ovule density is probably due to an effect of YPet–rgl1Δ17 in ovary shortening, rather than a direct effect in ovule primordia formation. In consequence, mature ovules in *YPet-rgl1Δ17* plants appeared to be closer to each other with folded or stretched funiculi that allow ovules to occupy less space within the ovary (Fig. 4B). Moreover, these ovules have severe alterations in morphology, mainly the total or partial loss of the embryo sac. Interestingly, normal and altered ovules were present side-by-side in the same pistil, without bias towards any particular ovary region (apical or basal). This phenomenon is further examined in the next section.

Mature *YPet-rgl1Δ17* plants showed a strong reduction in fertility, with fruits that were much shorter than those in L*er* (Fig. 4C). When quantified, seed number was reduced by 60% when compared with a control L*er* plant (Fig. 4D). Reduced fruit size may be a direct consequence of reduced seed content, but also to the blockage of valve elongation during silique development.

The mild reduction in ovule number was not the major cause for reduced fertility in *YPet-rgl1Δ17* plants (Fig. 4A, C). To understand whether the *YPet-rgl1Δ*17 defect in seed-set was due to maternal and/or paternal causes, a reciprocal cross-pollination assay was carried out. For this, pistils of L*er* and *YPet-rgl1Δ17* plants were pollinated with either L*er* or *YPet-rgl1Δ17* pollen and the amount of seed set was determined. As shown in Fig. 4E, fertility defects in *YPet-rgl1Δ17* plants were of maternal origin. Fruits from L*er* plants pollinated with either L*er* or *YPet-rgl1Δ17* pollen produced a similar number of seeds. In contrast, pistils from *YPet-rgl1Δ17* plants always produced fewer seeds, regardless of the pollen origin (L*er* or *YPet-rgl1Δ17*). Although expression of RGL1 in *YPet-rgl1Δ17* plants was also detected in anthers (Fig. 3E, F), no significant defects in pollen were observed, as fertility was identical between fruits pollinated with either L*er* or *YPet-rgl1Δ17* pollen regardless of the pistil genotype.

Similar to *Ypet-rgl1Δ17*, plants expressing *YPet-rgl2Δ17* also had reduced fertility, but here this was caused mainly by defects in stamen development (Gomez *et al*., 2019). Therefore, both lines are essential to uncover the differential roles of RGL1 and RGL2 in fertility: whereas RGL1 has a major role in maternal fertility and pistil/silique elongation, RGL2 is a major player in male fertility, with only a marginal role in silique elongation.

### RGL1 impairs embryo sac development

Fertility defects in *YPet-rgl1Δ17* plants were of maternal origin, but were not caused solely by the reduced ovule number (Fig. 4A), pointing to ovule defects as the major cause for the reduced seed-set (Fig. 4B). To get a deeper insight into the role of RGL1 in ovule development, ovules in *YPet-rgl1Δ17* plants were dissected by CLSM and light microscopy techniques.

**Fig. 5.**
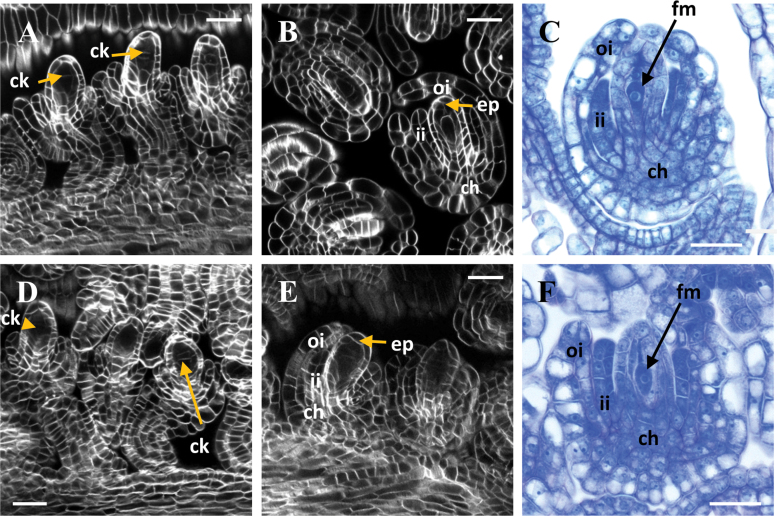
Ovule development was normal in *YPet-rgl1Δ17* plants until FM differentiation. Images of ovules of L*er* (A–C) and *rgl1Δ17* (D–F) plants at stages 2-V (A, D) and 3-I (B, C, E, F). Images (A, B, D, E) are CLSM, and images (C, F) are resin sections in light microscopy. Scale bars represent 20 µm in (A, B, D, E), and 50 µm in (C, F). ch, chalaza; ck, cytokinetic division (after meiosis); ep, nucellar epidermis; fm, functional megaspore; ii, inner integument; oi, outer integument. (This figure is available in colour at *JXB* online.)

**Fig. 6.**
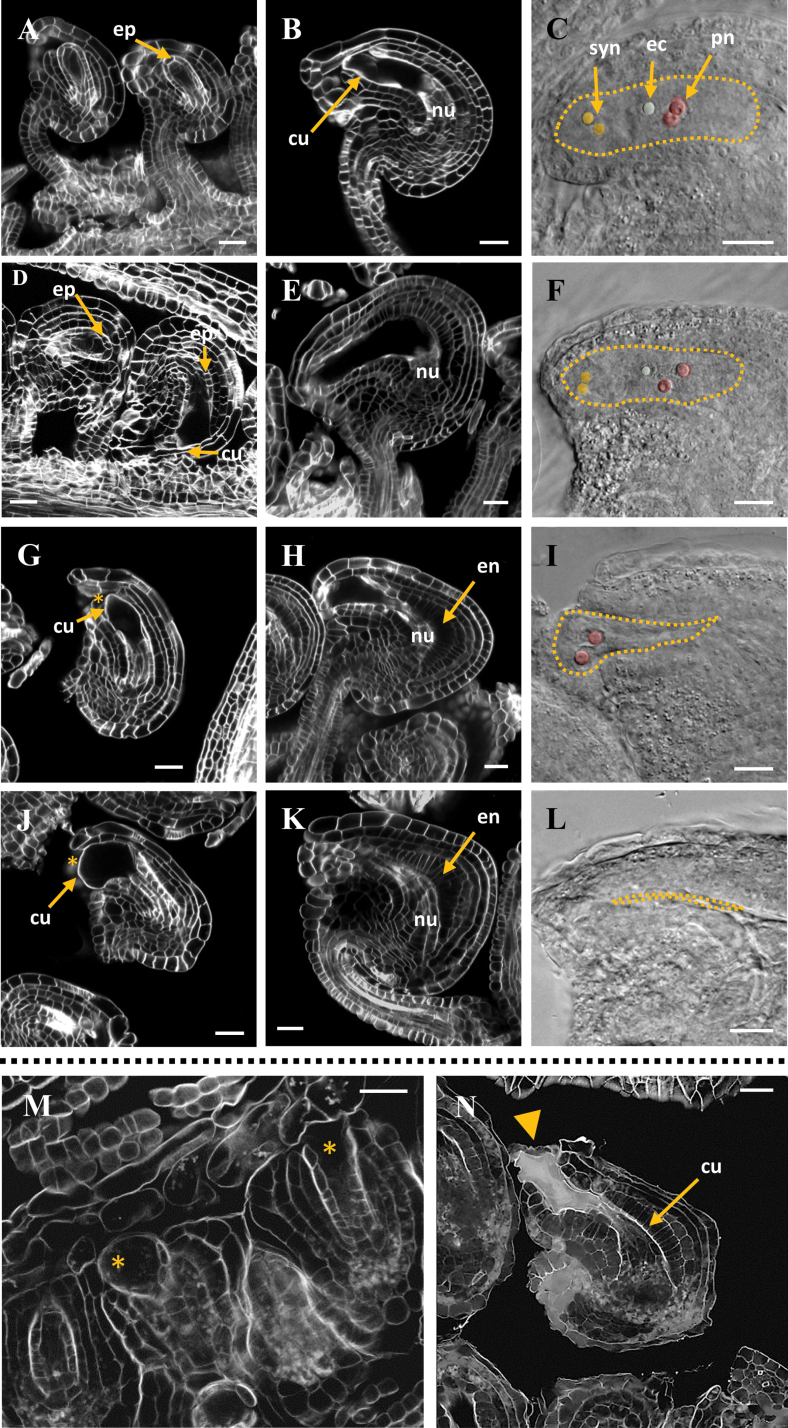
Embryo sac development is impaired in *YPet-rgl1Δ17* plants during megagametogenesis. (A–L) Images of ovules of L*er* (A–C) and *YPet-rgl1Δ17* (D–L) plants at stages 3-III (A, D, G), stage 3-IV (J), or mature ovules (B, C, E, F, H, I, K, L, N). (M, N) images of ovules of *YPet-rgl1Δ17* at stage 3-II (M) or mature ovule (N). In (D) a normal (left) and an abnormal (right) ovule is shown. Images were captured by CLSM, except (C, F, I, L), which were captured by differential interference contrast light microscopy. Scale bars represent 20 µm in all panels. Arrows in (D, G, J) point to the cuticle that separates inner integument and developing gametophyte. Asterisks in (G, J, M) mark the degenerated nucellar epidermis. Arrowhead in (N) points to embryo sac content being released from the ovule. In (C, F, I, L), dotted lines define the mature embryo sac, and synergids, polar nuclei, and the egg cell are colour-coded (as indicated in (C)). cu, cuticle layer; ec, egg cell; en, endothelium; ep, nucellar epidermis; nu, nucellar tissue; pn, polar nuclei; syn, synergids. The cuticle layer is auto-fluorescent. (This figure is available in colour at *JXB* online.)

Ovules in *YPet-rgl1Δ17* and L*er* developed similarly, both morphologically and temporally, until the formation of the FM (Fig. 5). Both L*er* and *YPet-rgl1Δ17* ovules showed cytokinesis marks inside the nucellus at stage 2-V (according to Schneitz *et al.*, 1995), indicating that meiosis of the MMC had occurred and tetraspores were formed (Fig. 5A, D). At stage 3-I, the three non-functional spores degenerated (Fig. 5B, E), and only the FM remained in L*er* and *YPet-rgl1Δ17* ovules (Fig. 5C, F). These observations indicate that the process of megasporogenesis occurred properly in *YPet-rgl1Δ17* plants. In contrast, from stage 3-I on, the embryo sac development was impaired (Fig. 6). We scored the number of altered ovules in *YPet-rgl1Δ17* plants and found that approximately 52% of mature ovules had a WT-like female gametophyte containing an egg, two polar, and two synergid nuclei (Fig. 6D–F), very similar to those in L*er* plants (Fig. 6A–C). The remaining 48% of *YPet-rgl1Δ17* ovules showed severe defects in embryo sac development (Fig. 6H, I, K, L). However, the percentage of altered ovules per pistil ranged approximately from 30 to 80%, showing a large range of penetrance of phenotype (see Supplementary Fig. S6). These defects were clearly visible at stage 3-III, pointing to a role for *YPet-rgl1Δ17* in altering the correct differentiation of the FM after stage 3-I, probably interfering with ovule development starting at the first mitotic division.

The defects in *YPet-rgl1Δ17* ovules were not homogeneous, since approximately 50% of the defective ovules retained a residual embryo sac (Fig. 6H, I) while the other 50% suffered a complete loss of the embryo sac (Fig. 6K, L). Therefore, the proportion of phenotypes among *YPet-rgl1Δ17* mature ovules was approximately 50% WT-like, 25% with mild defects, and 25% with severe defects (total loss of embryo sac). Moreover, the reduced embryo sac usually contained a smaller number of nuclei than L*er* ovules (Fig. 6I, L; compare with Fig. 6C), which impedes fertilization. In addition, we also observed ovule primordia with a premature loss of nucellar tissue in *YPet-rgl1Δ17* plants (stages 3-II and 3-III, see asterisks in Fig. 6M and arrows in Fig. 6D, G, J).

In Arabidopsis, the embryo sac growth displaces the nucellar tissue starting from the micropyle (Schneitz *et al.*, 1995). This process is clearly observable from stage 3-IV where the nucellar tissue is seen laterally (Fig. 6A). In L*er* mature ovules, the nucellus is nearly completely resorbed except for a group of cells at the base of the embryo sac (Fig. 6B). Upon resorption of the nucellus, a cuticle layer surrounds and separates the embryo sac from the inner integument (Fig. 6B) (Schneitz *et al.*, 1995; Beeckman *et al.*, 2000). The cuticle is an auto-fluorescent hydrophobic barrier formed by cutin, which later separates the maternal tissue from endosperm in fertilized ovules (Coen *et al.*, 2019). In *YPet-rgl1Δ17* plants, defective ovules showed a premature degradation of nucellar tissue, which led to alterations in embryo sac shape (Fig. 6G–L), or ovules with a fragile embryo sac cuticle that led to rupture and release of the content of the sac at stage 3-IV or 3-V, as observed in Fig. 6N. This event would explain the existence of mature *YPet-rgl1Δ17* ovules without an embryo sac or, instead, disorganized cell remains (Fig. 6K, L). It should be noted that *YPet-rgl1Δ17* ovules presented a characteristic triangular shape, especially pronounced in those without embryo sac, possibly due to an elongation of the cells of the endothelium (innermost layer of inner integument) (Fig. 6E, H, K).

So far, no evidence of similar defects in embryo sac development has been reported for other dominant mutants of *GAI*, *RGA*, and *RGL2*. As can be observed in Supplementary Fig. S7A–E, *gai-1*, *GFP-rgaΔ17*, and *YPet-rgl2Δ17* plants showed mature ovules with normal embryo sac. Moreover, a comparison of ovule and seed number in L*er* and all four dominant mutants confirmed that only *YPet-rgl1Δ17* showed a strong reduction of seed number, whereas ovule number was not reduced to the same extent (see Supplementary Fig. S7F). Finally, the loss-of-function mutant *rgl1-1* or a silenced line (rgl1D17-R) that behaves as a loss-of-function phenotype of RGL1 does not show defects in plant development, including fertility (Lee *et al.*, 2002; Wen and Chang, 2002).

As RGL1 acts as a repressor of embryo sac development, it would be expected that lack of RGL1 activity in *rgl1-1* should not result in any defect in ovule development. These data strongly suggest that the DELLA role in embryo sac development is RGL1-dependent and -specific. Our data clearly reveal that GAs have a role in the control of embryo sac development, which is mediated solely by RGL1. In *YPet-rgl1Δ17* plants, stable YPet–rgl1Δ17 should block downstream events essential for embryo sac formation, probably shortly after the first mitotic division of the FM.

### **Localization of YPet–rgl1**Δ**17 correlates to ovule defects**

**Fig. 7.**
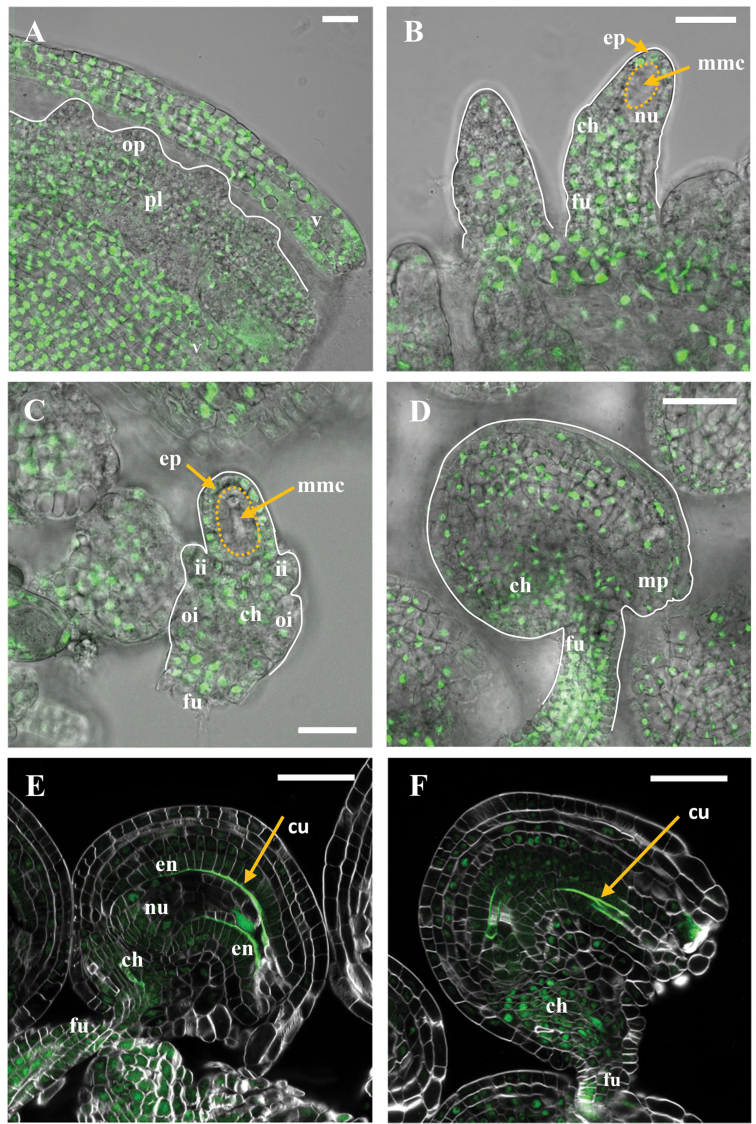
YPet–rgl1Δ17 expression during ovule development. CSLM images of *YPet-rgl1Δ17* developing ovules. (A) YPet–rgl1Δ17 was expressed in valve, placenta, and slightly in ovule primordia at stage 1-I. (B) Expression was detected in developing ovules at stage 2-II, in the funiculus, chalaza, and nucellar epidermis, but it was excluded from the megaspore mother cell (MMC) in the centre of the distal portion. (C) Expression appeared slightly in the integument primordia at stage 2-IV. (D) Expression in mature ovule epidermis (the outermost layer of outer integument). (E–F) YPet–rgl1Δ17 expression in abnormal mature ovules with remaining (E) or absent (F) embryo sac. Dotted lines in (B, C) define the MMC. Arrows in (E–F) point to the cuticle between the maternal and zygotic tissue. Scale bars represent 20 µm in (A–C) and 50 µm in (D–F). ch, chalaza; en, endothelium; ep nucellar epidermis; fu, funiculus; ii, inner integument; mmc, megaspore mother cell; mp, micropyle; nu, nucella; oi, outer integument; op, ovule primordia; pl, placenta; v, valve. The cuticle layer is auto-fluorescent. (This figure is available in colour at *JXB* online.)

As in the floral organs, RGL1–YPet protein was not detected during ovule development, and therefore the expression pattern of RGL1 was inferred by visualizing YPet–rgl1Δ17 protein by CSLM. The expression profile of *YPet-rgl1Δ17* correlates with ovule phenotypes (Fig.7; Supplementary Fig. S8). During early pistil development, *YPet-rgl1Δ17* was expressed at high levels in the pistil, valve, and placenta, and it was slightly detected in ovule primordia at very early stages of development (stage I-1) (Fig. 7A). Soon after, expression could be localized in the funiculus, chalaza, and nucellar epidermis of ovule primordia at stage 2-II, but it was excluded from the germline cell in the centre of the distal portion (Fig. 7B). *YPet-rgl1Δ17* expression increased in developing ovules and started to be detected in the integument primordia at stage 2-IV (Fig. 7C). Finally, expression was clearly detected in the mature ovule at anthesis (Fig. 7D). The protein localization data, obtained with the *YPet-rgl1Δ17* line, were supported by the expression of the *RGL1* gene during ovule development by *in situ* mRNA hybridization (Supplementary Fig. S9). To determine the expression of *YPet-rgl1Δ17* in different cell layers of mature ovules with defects in embryo sac development, cleared ovules were examined by CSLM (Fig. 7E). Expression was detected in the funiculus, chalaza, and endothelium layer, and in other integument cell layers at a lower level. Level of *YPet-rgl1Δ17* expression correlates to ovule defects (Supplementary Fig. S10); in WT-like ovules, expression was lower than in those with severe defects. The highly fluorescent layer between the endothelium and the impaired embryo sac corresponds to the cuticle (Fig. 7E, F). Cutin deposition was also detected in ovules in which no embryo sac was observed (Fig. 7F). As cutin deposition around the nucellus takes place upon mitosis of the FM, the presence of this layer in *YPet-rgl1Δ17* ovules with severe phenotypes suggests that these ovules underwent megagametogenesis and developed a weak embryo sac that later ruptured.

Taken together, expression and phenotype analysis indicate that *YPet-rgl1Δ17* affects embryo sac development from the neighbouring cells. In Arabidopsis, genetic studies have proposed that the development of the FM (megasporogenesis) and embryo sac (megagametogenesis) depends on information from surrounding diploid cells (Yang *et al.*, 2010; Lora *et al.*, 2016; Pinto *et al.*, 2019). Before the appearance of the integuments, *NOZZLE/SPOROCYTELESS* (*NZZ/SPL*) (Yang *et al.*, 1999) and *WUSCHEL* (*WUS*) (Lieber *et al.*, 2011) participate in coordination to regulate the differentiation of the MMC. Interestingly, these genes are expressed in the nucellar epidermis, but influence the haploid FM development, suggesting that they would act non-cell autonomously in the control of female germline progress.

For example, *NZZ*/*SPL* is required to regulate the expression of *PIN-FORMED 1*, an auxin efflux transporter, in the nucellar epidermis to modulate auxin fluxes to the MMC (Bencivenga *et al.*, 2012; Pinto *et al.*, 2019). Another example is *CYP78A5/KLUH* (*KLU*), a gene involved in chromosome pairing during female meiosis, although it is expressed at the base of the nucellus in the region initiating the inner integument. Possibly, *KLU* performs this function through the production of a mobile signal that diffuses from these tissues to the surrounding cells (Zhao *et al.* 2014). Moreover, analysis of a set of key genes necessary for integument development, which include *AINTEGUMENTA* (Klucher *et al.*, 1996), *INNER NO OUTER* (Villanueva *et al.*, 1999), *KLU* (Zhao *et al.*, 2014), and *BELL1*, *SEEDSTICK*, and *SHATTERPROOF 1* and *2* (Battaglia *et al.*, 2008), also supports non-cell-autonomous signalling. The phenotypes of the corresponding mutants demonstrate that these genes not only control integument identity but that they also play a role during megasporogenesis, since embryo sac maturation is impaired. Recently, it has been reported that the mis-expression of the transcription factor *FUSCA3* in the integuments severely impairs embryo sac development (Wu *et al.*, 2020). Finally, *ARGONAUTE5* (*AGO5*), an effector of small RNA (sRNA) silencing pathways, is required to promote megagametogenesis in the FM (Tucker *et al.*, 2012). *AGO5* is expressed in the inner integument and nucellar epidermis and is thought to participate in embryo sac development by transmitting an sRNA into the FM, repressing movement of a protein or metabolite from the nucellar epidermis or by indirectly influencing nucellus development. All this evidence suggests that inter-regional signalling is important during megagametogenesis.

The data shown here suggest that RGL1 protein could behave like these genes, specifically, like *AGO5*. Based on the effect of the GA-resistant YPet–rgl1Δ17 protein, we hypothesized that RGL1 activity alters proper embryo sac development after the megaspore has been developed, although it is only expressed in integuments and the nucellar epidermis. It is well known that the function of DELLA proteins, including RGL1, lies in their ability to establish protein–protein interactions with a multitude of regulatory proteins, mostly transcription factors (Davière and Achard, 2013, 2016). Upon binding, the DELLA modifies the DNA-binding capacity or the transcriptional activity of their interactor proteins. A plausible scenario is that RGL1 could bind and block a key transcription factor that is necessary for the correct development of the embryo sac, by impeding transcriptional activity towards its target genes. This mechanism has been well described previously for other developmental processes (Davière and Achard, 2013; 2016). For example, DELLA proteins interact with BRASSINAZOLE RESISTANT 1 (BZR1) to inhibit its DNA-binding ability, thereby blocking BZR1-mediated transcriptional activity during hypocotyl elongation (Bai *et al.*, 2012; Gallego-Bartolomé *et al.*, 2012; Li *et al.*, 2012). Therefore, during ovule maturation in WT plants, GAs must mitigate the action of RGL1 in integuments and the nucellus by promoting its degradation, via the ubiquitin–proteasome pathway, to allow adequate gametophyte development.

### Bioactive GAs are located in developing ovules

**Fig. 8.**
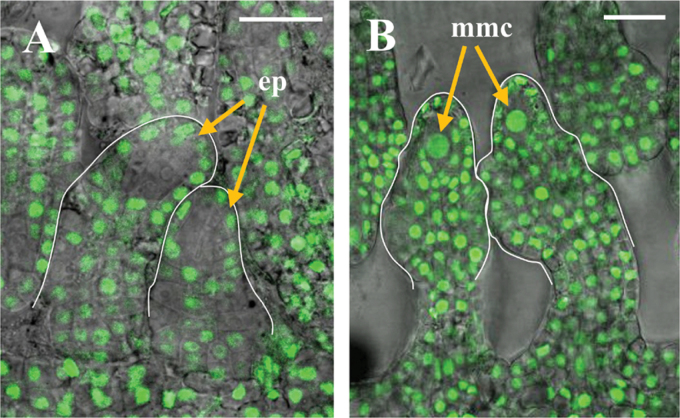
Localization of YPet–rgl1Δ17 and bioactive GAs in ovules. (A) YPet–rgl1Δ17 expression in ovules at stage 2-III. (B) Localization of bioactive GAs using the GA HACR reporter signal (Khakhar *et al.*, 2018) in ovules at stage 2-III. Scale bars represent 20 µm. ep, nucellar epidermis; mmc, megaspore mother cell. (This figure is available in colour at *JXB* online.)

The abnormal embryo sac development observed in *YPet-rgl1Δ17* plants is probably the result of the RGL1-dependent blockage of the normal developmental programme that the megaspore undergoes during ovule development. Therefore, in normal ovules, GAs would be present in the developing ovule to degrade RGL1 (and probably other DELLA proteins) and allow normal growth and development. To visualize the presence of bioactive GAs in the ovule primordia, we used plants transformed with a GA sensor (GA HACR) based on the GA-sensitive RGA that targets a Venus reporter protein (Khakhar *et al.*, 2018). In these plants, endogenous bioactive GA distribution is visualized as a Venus fluorescence signal in confocal microscopy. At stage 2-III of ovule development, fluorescence could be observed in the large central nucleus of the megaspore mother cell, and in the surrounding tissues (Fig. 8A), including the nucellar epidermis, where RGL1 was also detected (Fig. 8B). So far, this is the first observation of active GAs inside the ovule primordia, which supports the participation of GAs in ovule development.

## Conclusions

**Fig. 9.**
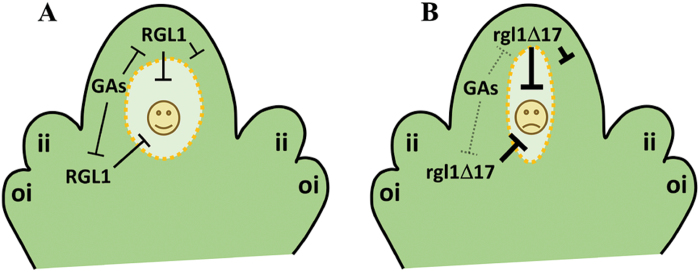
Working model of the role of RGL1 in the control of embryo sac development. GAs were detected through the ovule primordia development, including the nucellar epidermis, integuments, and the MMC. YPet–rgl1Δ17 was located in the nucellar epidermis and integuments, but not in the germline. RGL1 represses correct development of the embryo sac and locally affects the nucellar epidermis. (A) In L*er* plants, GAs in the nucellar epidermis and integuments mediated the degradation of endogenous RGL1, which allows the correct megagametogenesis. (B) In contrast, in *YPet-rgl1Δ17* plants stable RGL1 protein was not degraded, impairing embryo sac development and altering nucellar epidermis. Weakening of the nucellar epidermis provoked the total or partial release of the embryo sac content. ii, inner integument; oi, outer integument. (This figure is available in colour at *JXB* online.)

Taken together, the data reported here uncover a new role of GAs in the coordinated control of ovule development, in particular the events that take place from the first rounds of mitotic division of the FM, and allow us to propose a working model (Fig. 9). RGL1 specifically represses normal development of the FM, as the GA-resistant YPet–rgl1Δ17 protein in the nucellar epidermis and integuments caused a partial or complete ablation of the embryo sac. On the other hand, bioactive GAs are detected throughout the ovule primordia development, including the nucellar epidermis and the MMC. In L*er* plants (Fig. 9A), GAs mediate the degradation of endogenous RGL1, which allows the correct megagametogenesis. In contrast, in *YPet-rgl1Δ17* plants (Fig. 9B), stable YPet–rgl1Δ17 protein is not degraded, impairing embryo sac development. Finally, YPet–rgl1Δ17 may also have a local effect in the nucellar epidermis, causing a weakening of epidermal cells that facilitates the release of the embryo sac content, visible in ovules with severe defects. Further studies should be carried out to find out exactly at what point RGL1 alters megagametogenesis. Regardless of this, our data suggest that RGL1 in the integuments and nucellar epidermis regulates genes involved in the progression of the FM mitotic cycle, nuclear positioning inside the embryo sac, expansion of the central vacuole, or the final cellularization, including proper nucellar epidermis degradation, processes that are necessary for correct megagametogenesis and embryo sac maturation. The identification of the *RGL1* target genes and interactors during megagametogenesis would be key to unravel the molecular mechanism underlying the role of GAs in the control of ovule development.

## Supplementary data

Supplementary data are available at *JXB* online.

Fig. S1. Scheme of the construction of *pRGL1:RGL1-YPet* and *pRGL1:YPet-rgl1Δ17* lines by recombineering strategy.

Fig. S2. Detailed scheme of the generation *pRGL1:RGL1-YPet* construct from YAtY clone JAtY50E24.

Fig. S3. Detailed scheme of the generation *pRGL1:YPet-RGL1* construct from YAtY clone JAtY50E24.

Fig. S4. Detailed scheme of the 17-aa deletion of the DELLA domain in *pRGL1:YPet-RGL1* construct.

Fig. S5. Detailed scheme of the final trimming of modified JAtY50E24 clones.

Fig. S6. Variable penetrance of embryo sac defects in pistils of *YPet-rgl1Δ17*.

Fig. S7. Defects in ovule development are specific to *YPet-rgl1Δ17*.

Fig. S8. YPet–rgl1Δ17 expression during ovule development.

Fig. S9. *In situ* RNA hybridization shows that *RGL1* is expressed in ovules during development.

Fig. S10. Correlation of the level of expression of *YPet-rgl1Δ17* with ovule phenotype.

eraa395_suppl_Supplementary_File001

## Data Availability

The data and material supporting the findings of this study are available from the corresponding author (MAP-A) upon request.

## Abbreviations

CaMV: cauliflower mosaic virus
CSLM: confocal scanning laser microscopy
FM: functional megaspore
GA: gibberellin
L*er*: Landsberg *erecta*
MMC: megaspore mother cell
