# Supplementary material for: Gibberellin-mediated RGA-LIKE1 degradation regulates embryo sac development in Arabidopsis
Source: J Exp Bot. 2020 Aug 26;71(22):7059–72. doi: 10.1093/jxb/eraa395 (PMC7906783; doi:10.1093/jxb/eraa395)

# Supplemental Figure S1

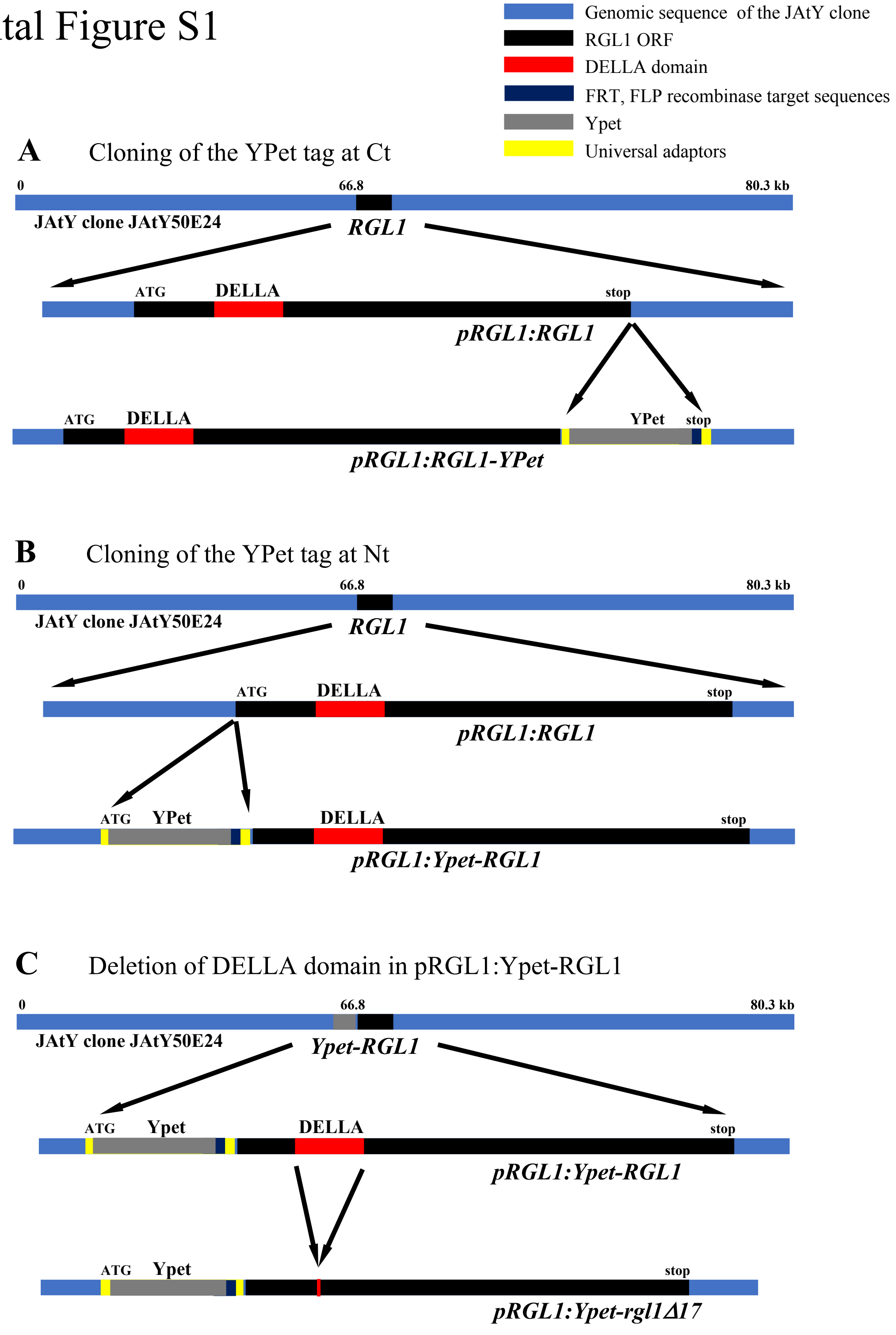


**Supplemental Figure S1.** Scheme of the construction of *pRGL1:RGL1-YPet* and *pRGL1:YPet-rgl1Δ17* lines by recombineering strategy from the JAtY50E24 clone.
(A) Cloning of the YPet tag at the Ct of *RGL1* locus. (B) Cloning of the *YPet* tag at
(B) the Nt of *pRGL1* locus. (C) Generation of the 17-aa DELLA domain deletion in
(C) RGL1 from the *pRGL1:YPet-pRGL1* construct.
(D) The different DNA fragments used are indicated.

# Supplemental Figure S2

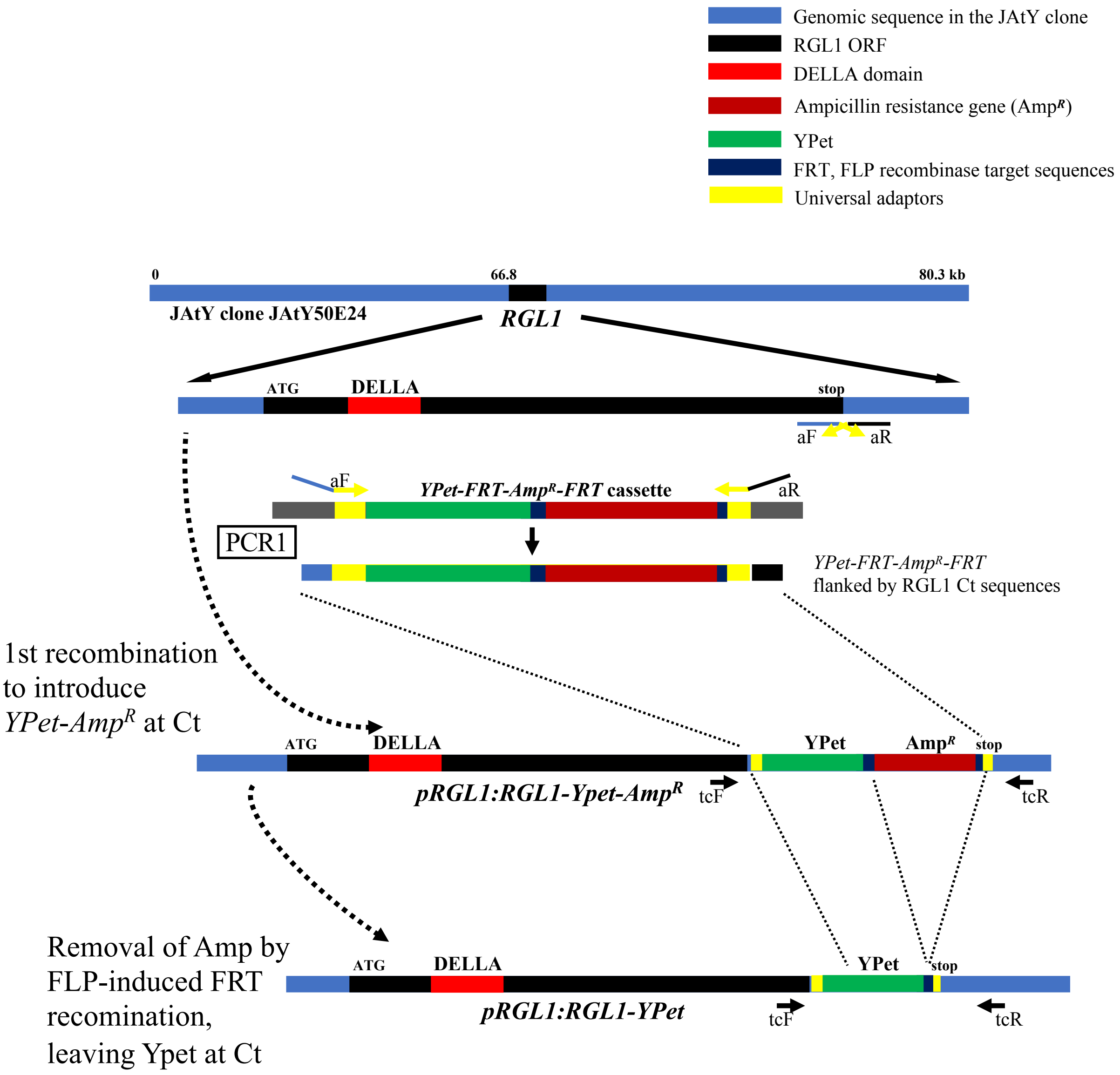


**Supplemental Figure S2.** Detailed scheme of the generation *pRGL1:RGL1-YPet* construct from YAtY clone JAtY50E24. The different DNA fragments used are indicated.

# Supplemental Figure S3

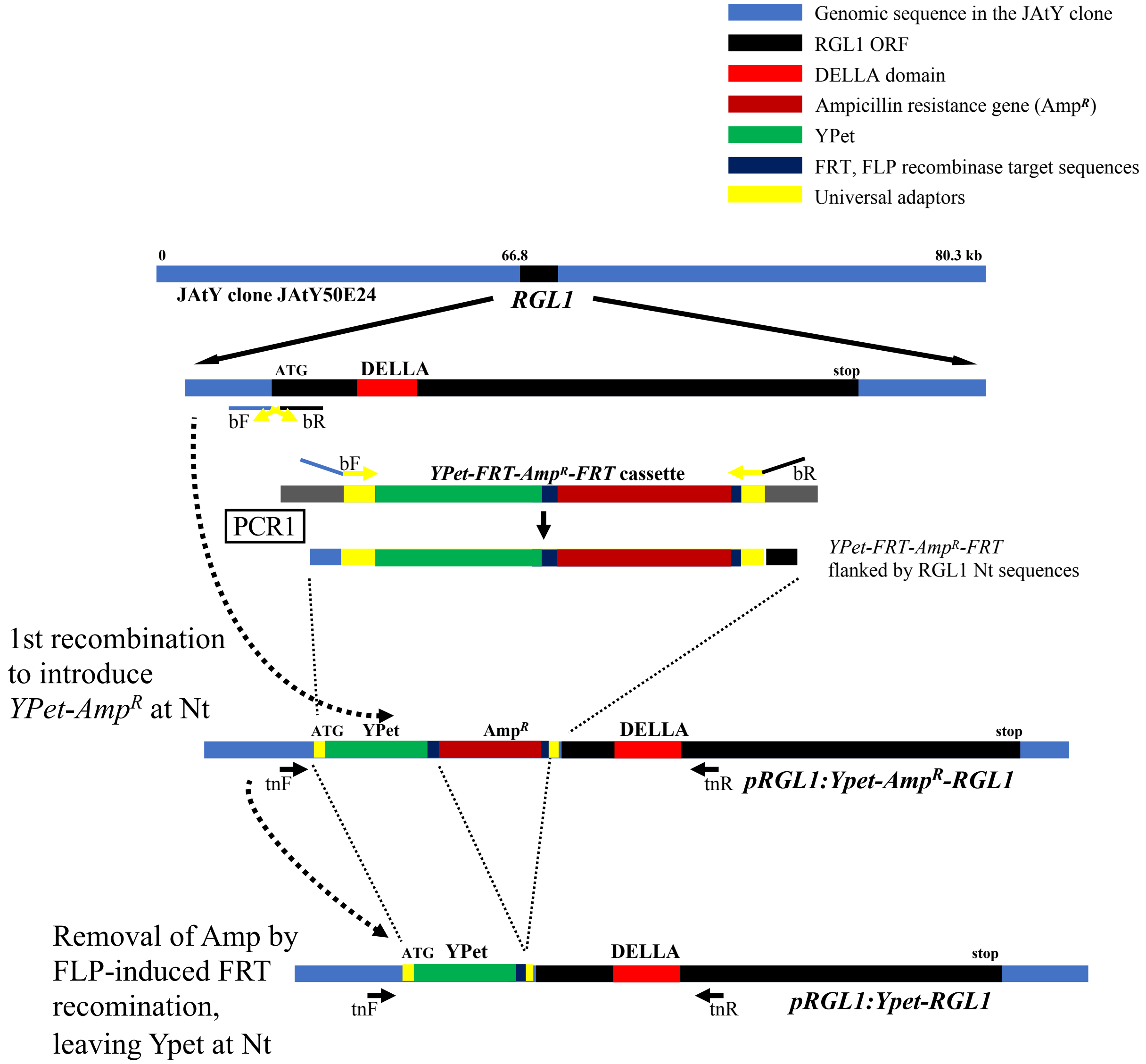


**Supplemental Figure S3.** Detailed scheme of the generation *pRGL1:YPet-RGL1* construct from YAtY clone JAtY50E24. The different DNA fragments used are indicated.

# Supplemental Figure S4

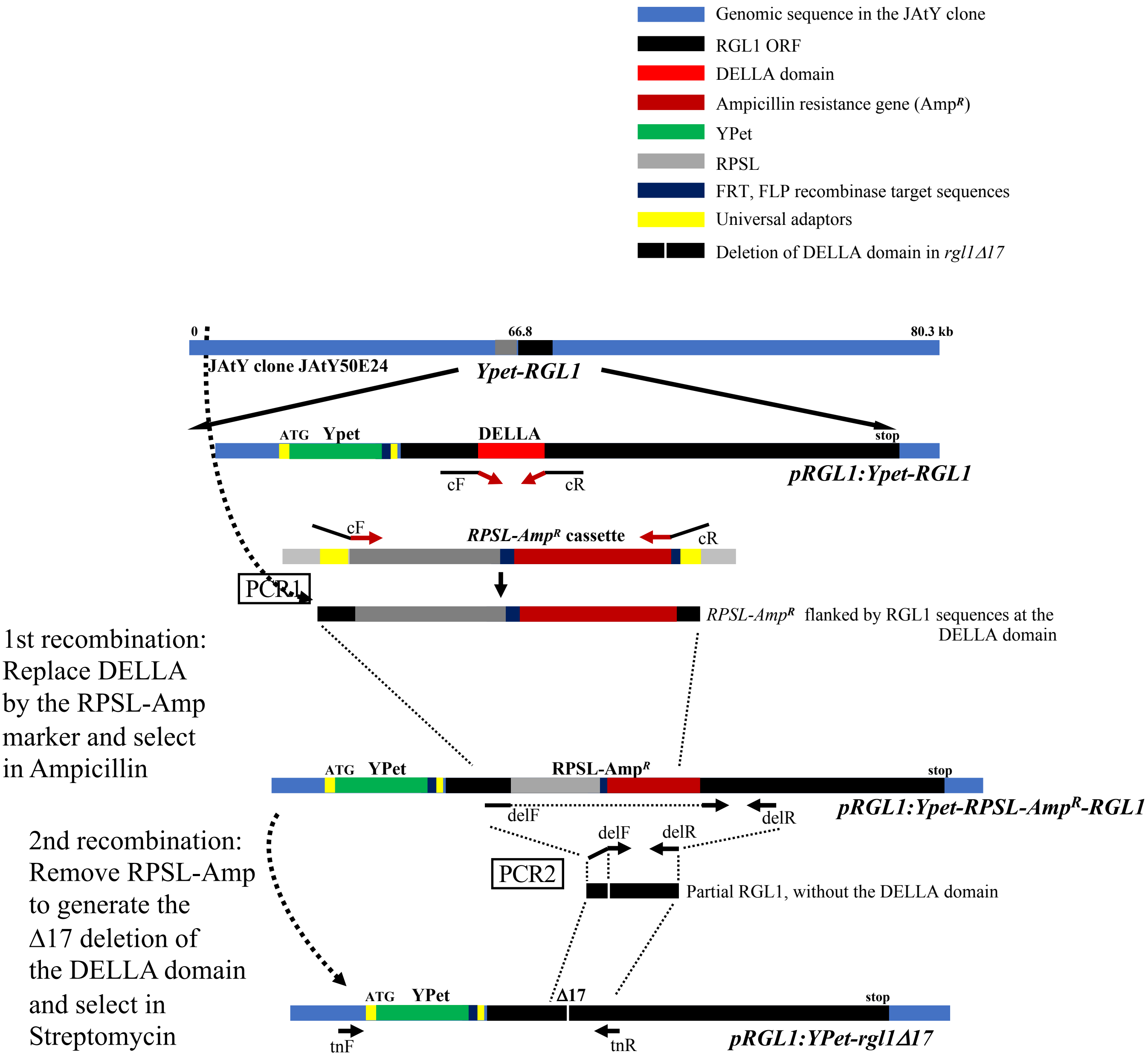


**Supplemental Figure S4.** Detailed scheme of the 17-aa deletion of the DELLA domain in *pRGL1:YPet-RGL1* construct. The different DNA fragments used are indicated.

# Supplemental Figure S5

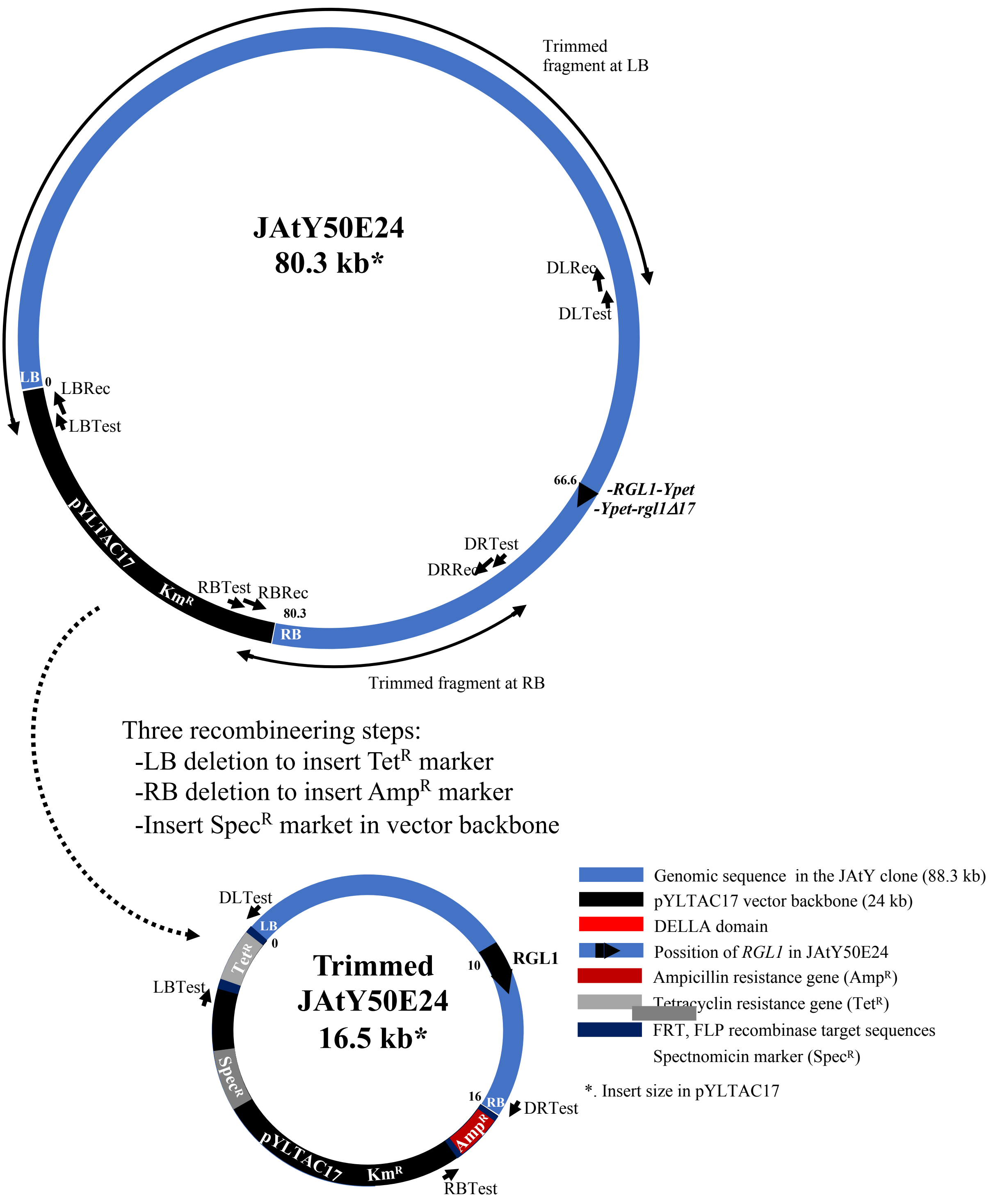


**Supplemental Figure S5.** Detailed scheme of the final trimming of modified JAtY50E24 clones to reduce the insert size from 80.3 kb to 16.5 kb, which included the *RGL1* locus and 10 kb and 5 kb upstream and downstream, respectively. A Spec$^{R}$ tag was also inserted in the vector backbone. Trimming and Spec$^{R}$ tagging were done in both *pRGL1:RGL1-YPet* and *pRGL1:YPet-rgl1Δ17* constructs. The different DNA fragments used are indicated.

# Supplemental Figure S6

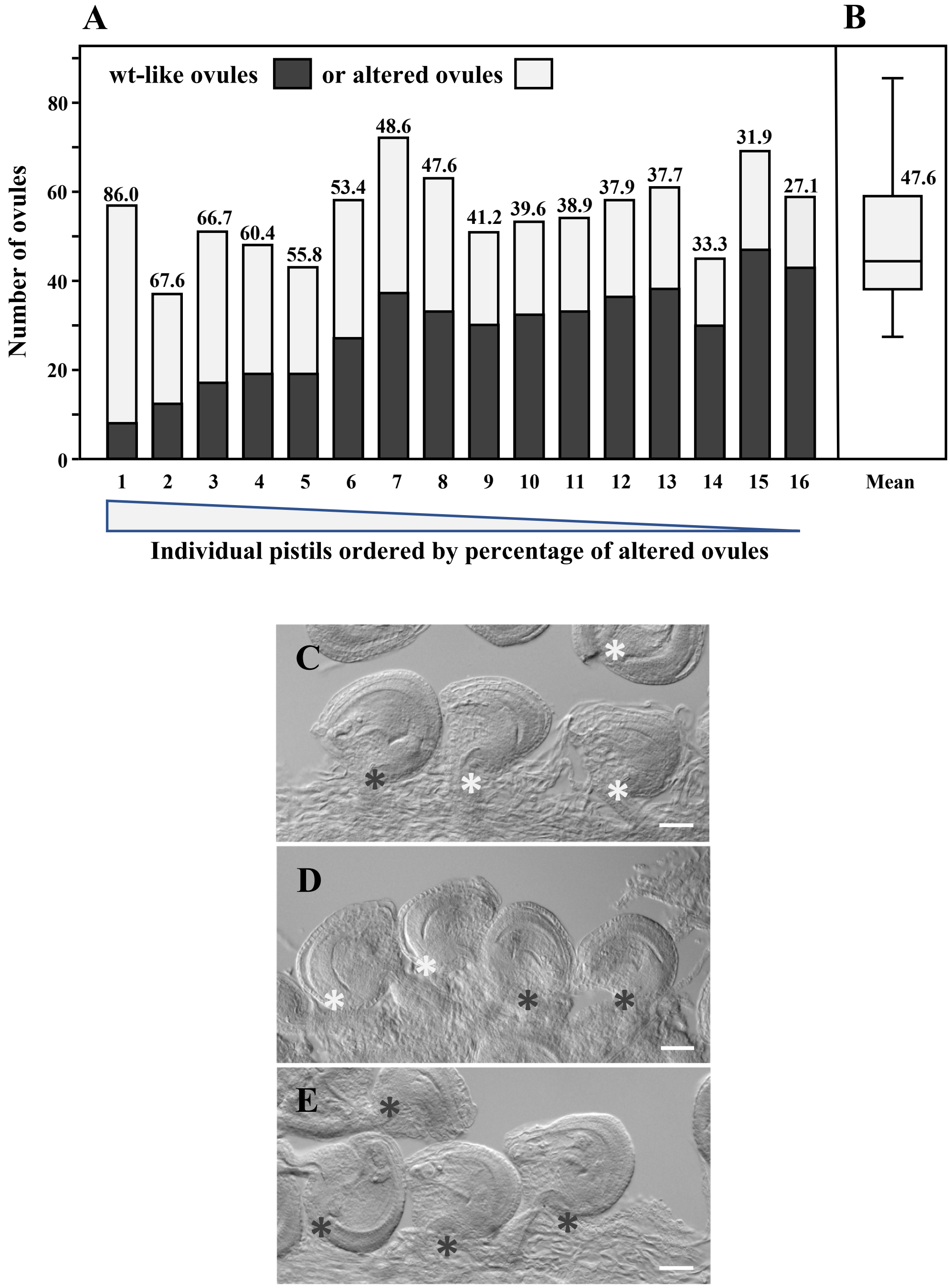


**Supplemental Figure S6.** Variable penetrance of embryo sac defects in pistils of *YPet-rgl1Δ17*. (A). Bars represent the number of wt-like ovules (dark grey) or ovules with defects in embryo sac (light grey) in 16 individual pistils. The percentage of ovules with embryo sac defects is shown above each column. (B) Average percentage of ovules with embryo sac defects, represented as boxplots, (n=875 ovules). (C-E) Representative images of pistils with high (C), medium (D), or low (E) percentage of defective ovules. Dark grey and light grey asterisks indicate wt-like or altered ovules, respectively. Scale bars represent 50 µm.

# Supplemental Figure S7

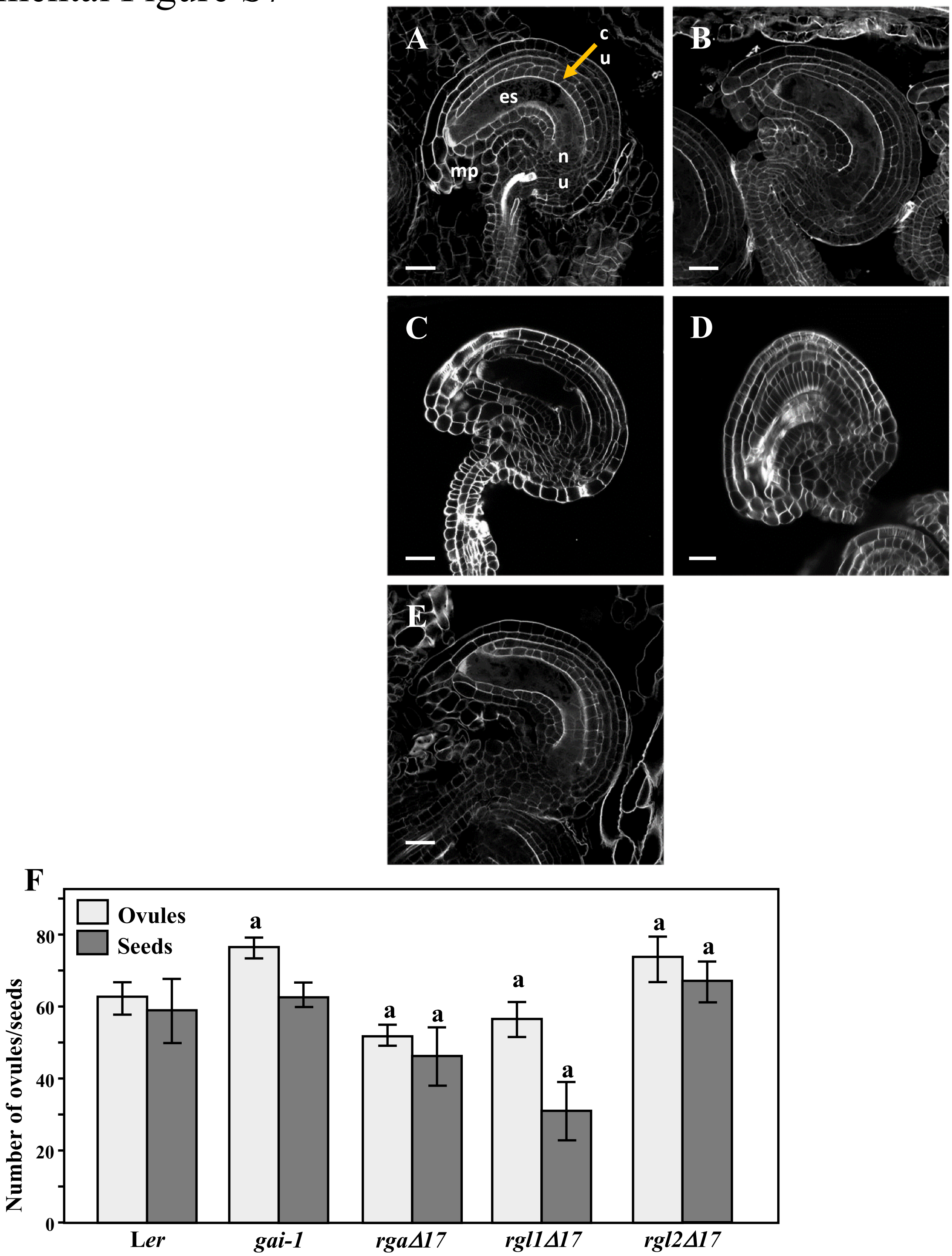


**Supplemental Figure S7.** Defects in ovule development are specific of *YPet-rgl1Δ17*. (A-E) CSLM images of mature ovules of L*er* (A) and dominant DELLA mutants, *gai-1* (B), *GFP-rgaΔ17* (C), *YPet-rgl1Δ17* (D), and *YPet-rgl2Δ17* (E). Scale bars represent 20 μm. In (A) the arrow points to the cuticle layer. Cu, cuticle layer; es, embryo sac; nu, nucellar tissue; mp, micropyle. (F) Ovule (light grey) and seed (dark grey) number in L*er* and *gai-1*, *GFP-rgaΔ17*, *YPet-rgl1Δ17*, and *YPet-rgl2Δ17* mutants. Significant differences (Student's *t*-test analysis) between L*er* and dominant *della* mutants are marked (a, *p*-value <0.01), and data shown are the mean and SD (n≥10). The cuticle layer is auto-fluorescent.

# Supplemental Figure S8

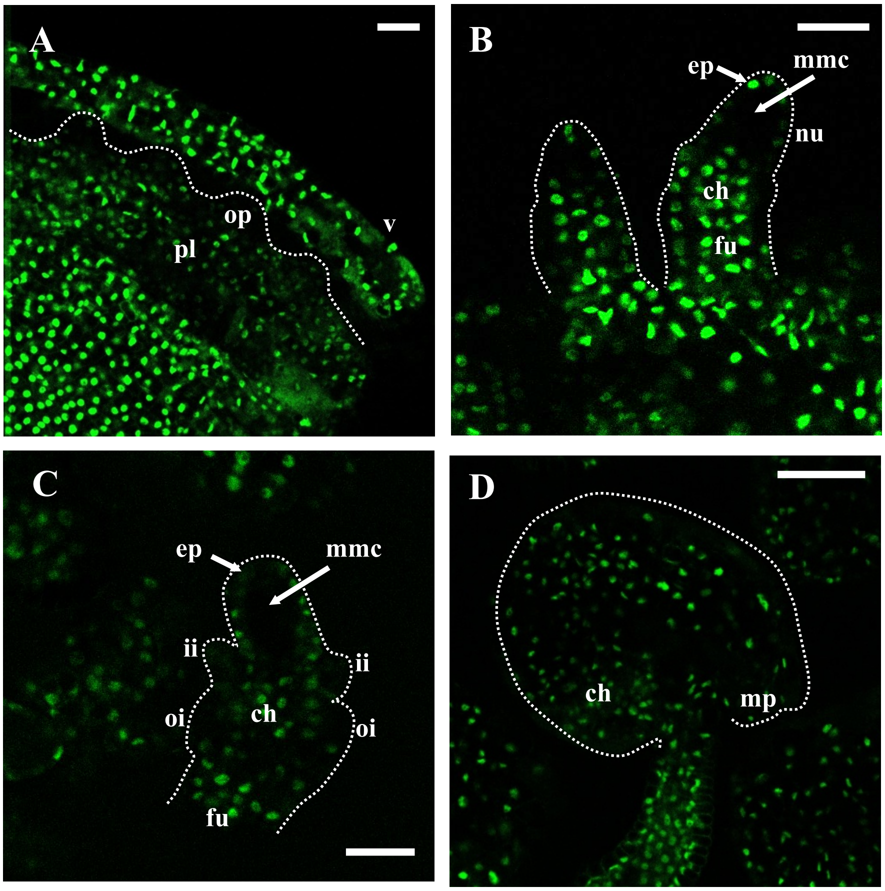


**Supplemental Figure S8.** YPet-rgl1Δ17 expression during ovule development. CSLM images of *YPet-rgl1Δ17* developing ovules from Figure 7, showing only the YPet fluorescence channel. Dotted lines define the ovule shape. Scale bars represent 20 µm in (A–C) and 50 µm in (D). ch, chalaza; ep nucellar epidermis; fu, funiculus; ii, inner integument; mmc, megaspore mother cell; mp, micropyle; nu, nucella; oi, outer integument; op, ovule primordia; pl, placenta; v, valve.

# Supplemental Figure S9

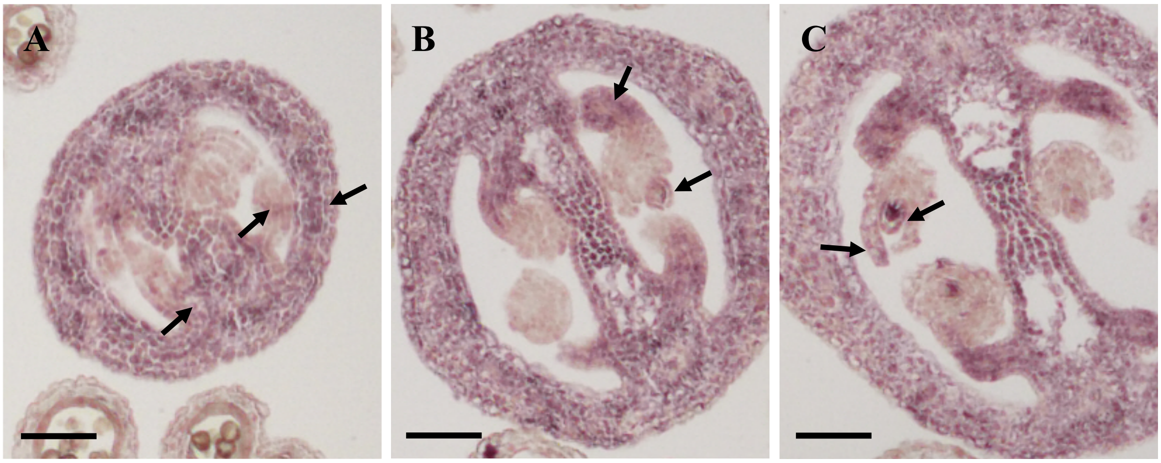


**Supplemental Figure S9.** *In situ* RNA hybridization shows that *RGL1* is expressed in ovules during development. Expression of *RGL1* in ovules at stages 2-II (A), 2-IV (B) and 3-II (C). In (A) arrows point to expression in valve, funiculus and chalaza; in (B) to funiculus and nucellus; and in (C) to nucellus and integuments. Scale bars represent 50 µm.

# Supplemental Figure S10

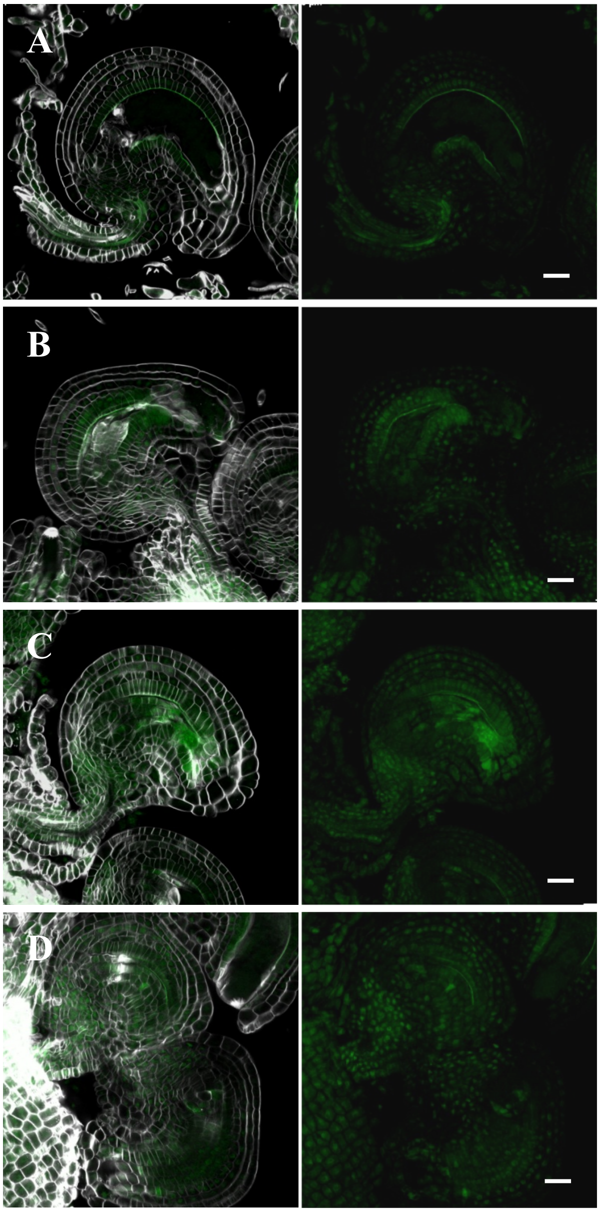

Supplement: eraa395_suppl_Supplementary_File001 [file eraa395_suppl_supplementary_file001.pdf]
